# A stochastic model for retinocollicular map development

**DOI:** 10.1186/1471-2202-5-30

**Published:** 2004-08-31

**Authors:** Alexei A Koulakov, Dmitry N Tsigankov

**Affiliations:** 1 Cold Spring Harbor Laboratory, One Bungtown Road, Cold Spring Harbor, NY, 11724, USA

## Abstract

**Background:**

We examine results of gain-of-function experiments on retinocollicular maps in knock-in mice [Brown et al. (2000) Cell 102:77]. In wild-type mice the temporal-nasal axis of retina is mapped to the rostral-caudal axis of superior colliculus. The established map is single-valued, which implies that each point in retina maps to a unique termination zone in superior colliculus. In homozygous Isl2/EphA3 knock-in mice the map is double-valued, which means that each point on retina maps to two termination zones in superior colliculus. This is because about 50 percent of cells in retina express Isl2, and two types of projections, wild-type and Isl2/EphA3 positive, form two branches of the map. In heterozygous Isl2/EphA3 knock-ins the map is intermediate between the homozygous and wild-type: it is single-valued in temporal and double-valued in the nasal parts of retina. In this study we address possible reasons for such a bifurcation of the map.

**Results:**

We study the map formation using stochastic model based on Markov chains. In our model the map undergoes a series of reconstructions with probabilities dependent upon a set of chemical cues. Our model suggests that the map in heterozygotes is single-valued in temporal region of retina for two reasons. First, the inhomogeneous gradient of endogenous receptor in retina makes the impact of exogenous receptor less significant in temporal retina. Second, the gradient of ephrin in the corresponding region of superior colliculus is smaller, which reduces the chemical signal-to-noise ratio. We predict that if gradient of ephrin is reduced by a genetic manipulation, the single-valued region of the map should extend to a larger portion of temporal retina, i.e. the point of transition between single-and doulble-valued maps should move to a more nasal position in Isl2-EphA3 heterozygotes.

**Conclusions:**

We present a theoretical model for retinocollicular map development, which can account for intriguing behaviors observed in gain-of-function experiments by Brown et al., including bifurcation in heterozygous Isl2/EphA3 knock-ins. The model is based on known chemical labels, axonal repulsion/competition, and stochasticity. Possible mapping in Isl2/EphB knock-ins is also discussed.

## Background

Topographic ordering is an important feature of the visual system, which is conserved among many visual areas [1]. Thus, the projection from retina to superior colliculus (SC) is established in a way, which retains neighbourhood relationships between neurons [2-4]. This implies that two axons of retinal ganglion cells (RGCs), which originate from neighbouring points in retina, terminate proximally in SC. It is assumed that this facilitates visual processing, which involves wiring local to the termination zone [5].

**Figure 1:**
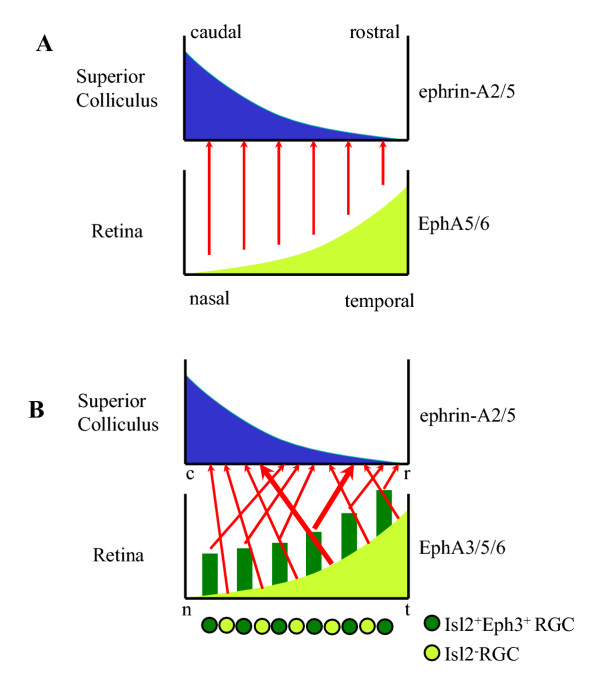
**Chemical labelling system in retinocollicular map in mice A. **Formation of the map in the wild-type mouse. Retinal ganglion cells (RGC) express EphA5/6 receptors in temporal > nasal gradient (bottom), whereas the cells in SC express the ephrin-A ligands in caudal > rostral gradient (top). Since axons of Eph+ RGC (red arrows) are repelled by ephrins this distribution of chemical markers leads to establishing of ordered topographic map in which nasal/temporal retina projects to caudal/rostral SC. This is because RGC axons expressing highest levels of Eph receptors (temporal) experience the largest repulsion and are expelled to the rostral part of SC, where such repulsion is minimal. Axons of nasal RGC are more indifferent to the action of ligands and occupy more caudal positions. Such system allows positioning of RGC axons in the order of increasing expression level of EphA receptors. **B. **Map in the mutant mouse from Ref. [20]. The expression level of EphA receptors was artificially increased in every second cell by genetic manipulations (dark gray). This is done by co expressing EphA3, which is absent in the wild-type RGCs (see **A**), with another gene, Isl2, which is expressed roughly in 50% of RGCs. Since ephrin ligands bind and activate all receptors from EphA family, albeit with different affinity, this results in anomalous projection to SC, based roughly on the total levels of EphA in each axon. Similarly to **A **this leads to sorting of axons in the order of increasing density of EphAs (red arrows). Note that two RGC neighboring in retina become separated in SC (bold arrows). This aberration in the topographic map leads to two termination zones (TZs) in SC for two neighboring cells in retina, rather than a single zone in wild-type [20].

The mechanisms responsible for topographic ordering have been lately under thorough examination. Following the original suggestion by Sperry [6], it was shown that chemical labels play an essential role in formation of the map (reviewed in [3,7]). For the projection from retina to SC the Eph family of receptor tyrosine kinases and their ligands ephrins were shown to be necessary for establishing correct topographic maps [7-10]. The coordinate system is encoded chemically in retina through graded expression of the Eph receptors by the RGCs. Thus, in mouse retina, two receptors of the family, EphA5 and A6, are expressed in the low nasal – high temporal gradient [11-14]. The recipient coordinate system in the SC is established through high caudal – low rostral gradient of ephrin-A2 and A5 ligands [15]. Since RGC axons expressing EphA receptors are repelled by high levels of ephrin-A ligands this system of reciprocal gradients allows sorting of the projecting axons in the order of increasing density of receptors, whereby contributing to the formation of topographic map [10,15,16] (Figure 1A). Thus, the system of reciprocal gradients is involved in formation of topographic representation along the nasal-temporal axis, albeit some additional fine-tuning is provided by activity-dependent mechanisms [17-19].

**Figure 2:**
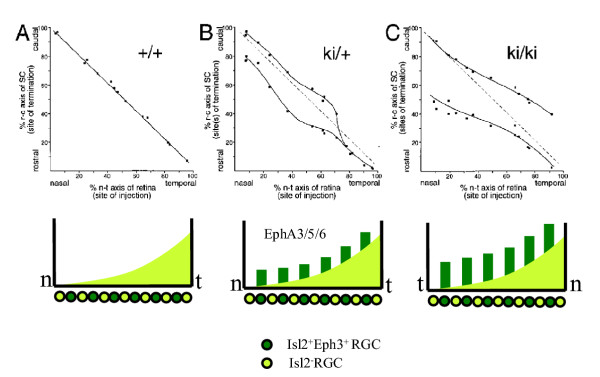
**Maps in Isl2+/Eph3+ mice. **The top row is reproduced from Ref. [20] (Figure 5). The bottom row illustrates the corresponding distribution of EphAs.

In this study we address the results of gain-of-function experiments, in which the retinocollicular maps were modified by genetic manipulations [20]. RGCs of the wild-type mouse express the LIM homeobox gene Islet2 (Isl2) [21]. Retina of a single animal is composed of two types of cells with regard to their expression of Isl2 gene, Isl2+ and Isl2-, which are intermixed in roughly equal proportion throughout the RGC layer (Figure 1B). To test the mechanisms of the retinocollicular map formation Brown et al. [20] generated "knock-in" mice, in which the Isl2 and EphA3 genes are coexpressed. This implies that each Isl2+ RGC and its axons, in addition to EphA5 and A6, also expresses EphA3, not found in the wild-type RGCs. The Isl2- cells remain EphA3-, as the wild-type cells. By doing so Brown et al. [20] increased the total level of EphA receptors in a given fraction of retinal cells. Since the overall level of EphAs is increased in Isl2+/EphA3+ cells, axons of two neighboring cells, knock-in and wild-type, should terminate in quite different places in SC (Figure 1B). The knock-in cells, interacting more strongly with the repellent should terminate at the position of decreased density of ephrins, i.e. more rostrally with respect to the wild-type cells. The neighborhood relationships between axons should be lost, the new map should lose its continuous nature, and it should split into two maps: one for wild-type RGCs, one for knock-in cells. This prediction was confirmed by experiments of Brown et al. [20] (Figure 2).

In addition to the observation of the overall map doubling in homozygous knock-ins (Figure 2C), Brown et al. discovered a curious behavior of the map in heterozygous animals. In these animals the exogenous levels of EphA3 were reduced roughly by a factor of two with respect to the homozygous knock-ins (Figure 2B). In terms of the expression density of EphA3 these animals stand between the wild-type and knock-in animals. Accordingly, the structure of the map resembles a hybrid of the wild-type and homozygous maps. The more rostral part of the map is single-valued, similarly to the wild-type, whereas about 60% of the caudal-most part is double-valued, like in the homozygous animals. This observation suggests that the map bifurcates somewhere between double-and single-valued regions. Although overall doubling of the map in homozygotes is easy to understand, any true model for the retinocollicular map formation should be able to account for the bifurcating behavior of map in heterozygotes. Therefore, experiments in heterozygotes represent a powerful tool to falsify various theoretical models.

Brown et al. [20] suggest that the bifurcating behavior of the map is consistent with the importance of relative rather than absolute values of the expression levels. Indeed, the relative difference of exogenous EphA3 to endogenous EphA5/6 is maximal in nasal retina (caudal SC), where the doubled map is observed (Figure 2B). In the temporal retina (rostral SC) the EphA3 to EphA5/6 ratio is not so large, which may account for the fact that the map is single-valued there. Thus a model for the topographic map from retina to SC should rely on the relative but not absolute levels of EphA signaling.

The point, which we make in this study, is that more experimental tests are needed to justify the suggestion about relative expression levels. To make our point clear we present a model for the retinocollicular map formation, which is based upon differences in the absolute values of Eph/ephrin expression levels, rather than relative differences. Our model manages to reproduce all the essential features of experiments described in Brown et al. [20], including bifurcation of the map in heterozygotes. In the model presented here the map is single-valued in rostral part of heterozygous SC due to inhomogeneous gradients of ligand and receptor, rather than reduced relative difference of EphA receptors. Below we suggest experimental tests, which may distinguish these two classes of models. This model was presented previously at Society for neuroscience meeting 2001 and on the arXiv preprint server [22,23].

To test various hypotheses we use a model for retinocollicular map formation employing stochastic Markov chain process. Our model is based upon three principles: chemoaffinity, axonal competition, and stochasticity. Some features of our model are similar to arrow model of Hope, Hammond, and Gaze [24]. The implementation of the model used here is available in [25].

## Results

### Markov chain model

**Figure 3:**
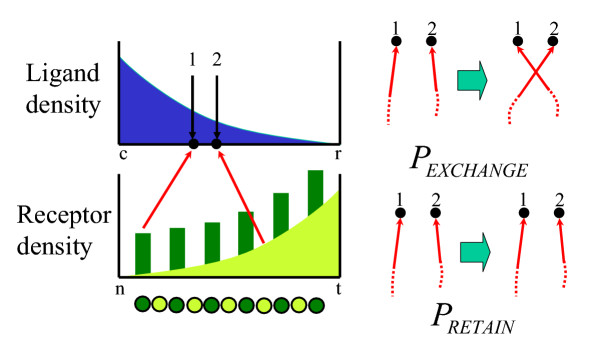
Description of the 1D model

Let us first describe the 1D version of the model. We consider a linear chain of 100 RGC, each expressing individual level of EphA receptors given by *RA*(*i*), where *i *= 1...100 is the RGC index, which also determines a discrete position of the cell in the retina. We have verified that results presented below do not depend on the number of cells, as long as this number is large enough. Each RGC is attached by an axon to one and only terminal cell in SC, which has an expression level of ligand given by *LA*(*k*), where *k *= 1...100 is the index in SC, also describing the terminal's topographic position. The receptor density *RA *is an overall increasing function of its index *i*, while the ligand density *LA *is decreasing, when going from *k *= 1 (caudal) to *k *= 100 (rostral) positions (Figure 3). This determines the layout of chemical "tags" used to set up map's "topography". An additional feature is that no two cells can project to the same spot in SC, which is meant to mimic axonal repulsion/competition for positive factors in SC, described in detail by Ref. [10].

We start from a random map, in which the terminal positions of all RGC axons in SC are chosen randomly. We then modify the map probabilistically, using the following rule. We consider two axons projecting to the neighboring points in SC (1 and 2 in Figure 3). We attempt to exchange these axons in SC with probability


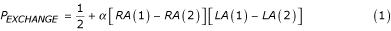


Here *α *> 0 is the parameter of our model. The probability of the axons to stay unchanged *P*_*RETAIN *_is determined from *P*_*EXCHANGE *_+ *P*_*RETAIN *_= 1 and is therefore given by


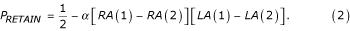


Since the only difference between these probabilities is the sign in front of *α*, it is important to describe the nature of this sign.

Assume that the product of gradients in Eq. (1) is negative, i.e. the gradients run in the opposite directions, which corresponds to the correct order of axonal terminals in SC. Then *P*_*EXCHANGE *_< 1/2 and *P*_*EXCHANGE *_<*P*_*RETAIN*_, i.e. the probability or retaining the current ordering of the axonal pair is larger than changing it. This is consistent with the chemorepellent interactions of receptors and ligands. In the opposite case of the wrong order, i.e. when the product of gradients in Eq. (1) is positive and gradients run in the same directions, *P*_*EXCHANGE *_>*P*_*RETAIN *_by the same reasoning. The described process will tend to exchange the order of gradients and, therefore establish the correct order of topographic projections. By using probabilities described by Eqs. (1) and (2) we incorporate the chemoaffinity principle into our stochastic model. This step is then repeated for another nearest neighbor couple, chosen randomly, and so on, until a stationary distribution of projections is reached. Such a process belongs to the class of Markov chain processes, since transformations to the map are determined only by the present state of the mapping and are not otherwise affected by development history [26].

**Figure 4:**
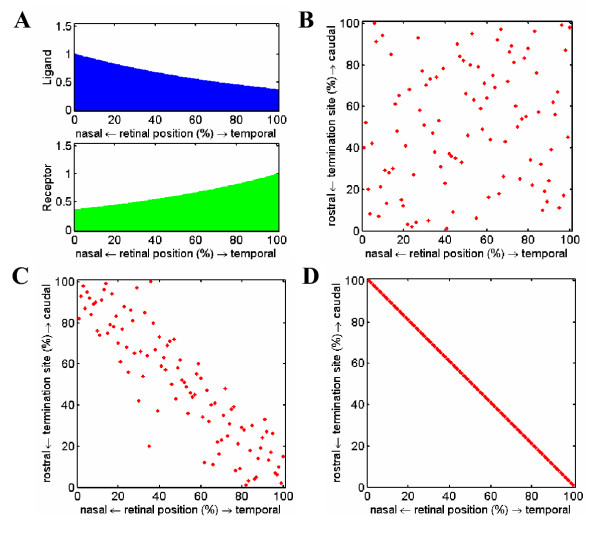
**Typical solutions of 1D model A**. Distribution of ligand (top) and receptor (bottom). **B**. Solution of the model for *α *= 0. Red dots represent terminal positions of individual axons, originating at various points in retina. If the case *α *= 0 the map is completely random, since all the chemical cues are multiplied by zero in Eq. (1), and, therefore, cannot contribute to the solution. **D**. *α *is very large. Solution represents perfect ordering of axons in SC in the order of increasing density of receptor. This is because the chemical cues are extremely strong in this case, much stronger than noise. **C**. *α *= 30. At the intermediate value of *α *solution is a compromise between chemical signal and noise.

Let us first consider cases in which final distribution can be understood without the use of computer. The model described by (1) can be solved exactly for at least two limiting cases: when *α *= 0 and when *α *is very large. In the former case (*α *= 0) the information about chemical labels cannot affect the solution, since it is multiplied by 0 in Eq. (1). Hence, the map is completely random (Figure 4B). In the latter case (*α *is large) the molecular cues are very strong. They eventually produce solution in which the axons are perfectly sorted in SC in the order of increasing density of receptor (Figure 4D). An intermediate situation with certain finite value of parameter *α *is described by a compromise between noise and chemical cues, with former randomizing the map on the finer scale, while the latter inducing the overall correct ordering (Figure 4C). We conclude that mean position of projections is controlled by the chemical signal, while the spread of projections or the size of TZ is determined by noise (Figure 4C).

It should be noted that in the case of large *α *(perfect sorting, Figure 4D) our model is equivalent to the arrow model, introduced by Hope, Hammond, and Gaze in [24]. The arrow model uses exchanges between nearest axons if they terminate the wrong way in tectum/SC. It can also include stochastic steps, as described in [24,27]. The stochastic behaviors of the model described here [Eqs. (1) and (2)] and the arrow model are not the same (see Discussion).

### Topographic maps in knock-in mice

**Figure 5:**
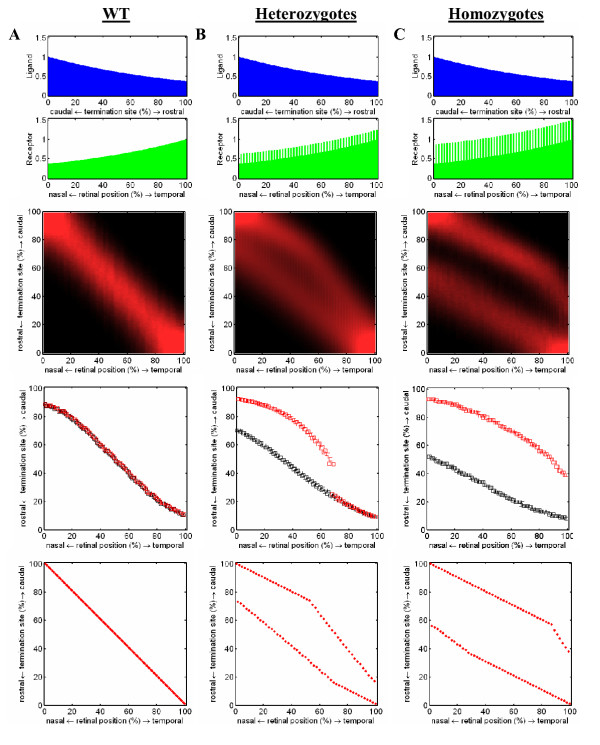
**1D maps in wild-type and knock-in animals. A**. column of results for wild-type conditions. **B**. Column corresponding to heterozygous Isl2/Eph3 knock-in conditions. The bifurcation of the probability distribution is similar to results of Ref. [20] (see Figure 2 above). **C**. Results for homozygous knock-in conditions. For all three conditions, the top row represents distributions of chemical labels. The second row shows the probability distributions. The brightness of color at each point in the middle image represents probability that an axon originating from the horizontal position projects to the point's vertical position. The bottom row shows positions of maxima of probability density distributions displayed above. The red squares correspond to wild-type cells; black markers determine maxima of distributions for Isl2+/EphA3+ RGCs. A slight even-odd oscillation observed in these panels is an artifact of ordering of wild-type and EphA3+ axons in retina. The bottom row shows maps obtained by sorting retinal receptor density.

Figure 5 summarizes results obtained in our model. The top row shows distributions of chemical 'tags' corresponding to the wild-type, heterozygous, and homozygous knock-in conditions in Brown et al. [20]. The second row shows the corresponding probability distributions for axonal projections. The third row in Figure 5 displays maxima of the probability distributions, shown to make map structure more visible. These results qualitatively agree with Brown et al. [20] (see Figure 2 above), for all three cases.

Maps in both wild-type mice and homozygote conditions (Figure 5, columns A and C) can be understood on the basis of axonal sorting in SC in the order of monotonously increasing levels of EphAs. Results of such sorting are displayed in the bottom row in Figure 5. It is clear that maps resulting from simple sorting reproduce all essential features of mapping observed in wild-type and homozygotes. At the same time, bifurcation, observed in heterozygotes (column B), is not captured by simple sorting procedure. This observation led us to develop the version of noisy sorting, based on chemical cues, described here.

Why does the blending of two maps occur in rostral SC? This question is addressed below in discussion section. Here we display manipulations with 'chemical tags', which can shift the bifurcation point in our model. These manipulations point to two factors, which contribute to the location of the bifurcation point. The first factor is inhomogeneous endogenous EphA5/6 density. It results in a smaller separation between two branches of the map observed in rostral SC (Figure 5B and 5C, bottom). The second factor is a smaller gradient of ligand in rostral SC (single-valued map region) than in caudal SC. Both these factors are addressed below.

**Figure 6:**
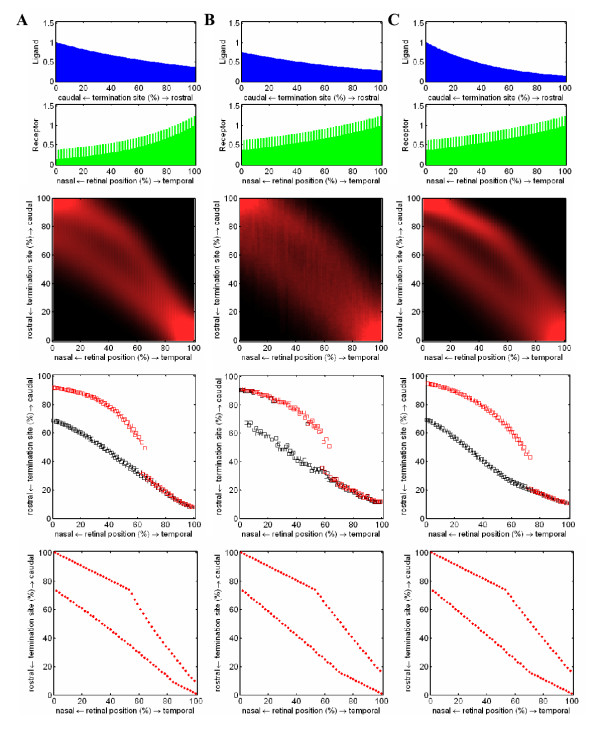
**Manipulations with chemical labels, which lead to shifts in the bifurcation point A. **The endogenous receptor gradient is made steeper compared to Figure 5B. The single-valued part of the map expands caudally. **B**. Density of ligand is reduced by 25%. Similar expansion of the single-valued part of the map is evident. **C**. The ligand gradient is made steeper. This is to reproduce the possible effect of ligand dimerization. The bifurcation point is moved slightly rostrally, expanding the double-valued part of the map. In all three columns the vertical arrangement of panels is similar to Figure 5.

Let us now demonstrate the impact of inhomogeneous EphA gradient on bifurcation. Figure 6 (column A) shows that if the endogenous gradient of receptor is made more inhomogeneous, the point of bifurcation is shifted caudally. This means that the single-valued part of the map becomes larger. To see this, notice that the density of receptor in Figure 5 (column B) has a minimum value of about 0.3. The minimum value of the receptor density in Figure 6 (column A) is about twice as small. Hence the endogenous receptor density in Figure 6A changes faster than in Figure 5. The results of simple sorting of axons according to increasing level of receptor are also shown in Figure 6A (bottom). Two branches of the map approach each other closer than in Figure 5B. This is consistent with the expanded single-valued part of the map observed in Figure 6A.

But is receptor distribution the sole determinant of the position of the bifurcation point? To demonstrate that the latter is also controlled by the gradient of ligand in SC we reduce the density of ligand uniformly by 25% (Figure 6B). This may mimic experiments in which increment in RGC receptor is combined with reduction in ephrin-A ligand density. As a result, the point of transition between single-valued and double valued parts of the map is located more caudally in Figure 6B than in Figure 5A. Hence, we can affect the point of transition by changing the densities of both receptor and ligand to a similar degree. These results are explained below in the discussion section.

Finally, we verify that increasing of the gradient of ligand leads to a small expansion of double-valued part of the map. This result is demonstrated in Figure 6C. This example shows once again that ligand concentration can affect the position of bifurcation point and that increasing the ligand profile inhomogeneity (Figure 6C, top) leads to a more pronounced bifurcation effect (compare to Figure 5B).

## Results for 2D model

We simulated 2D development using the hypothesis that another pair of chemical tags, EphB family of receptors and their ligands, ephrins-B, are responsible for establishing topographic projection from dorsal-ventral (DV) axis on retina to lateral-medial axis in SC [9]. EphB2/3/4 are expressed in high-ventral-to-low-dorsal gradient by RGCs [28-30], while ephrins-B are expressed in high-medial-to-low-lateral gradient in tectum/SC [30]. Since dorsal/ventral axons project to lateral/medial SC this implies attractive interactions between EphB+ axons and ephrin-B rich environment [31] (see, however [32]). In our model the attractive interactions are modeled by the following exchange probability of two axonal terminals in the DV direction:


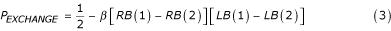


Here *RB*(1), *RB*(2), *LB*(1), and *LB*(2) are EphB receptor and ephrin-B ligand densities at neighboring points 1 and 2 in SC. This probability is similar to Eq. (1). Notice a sign change compared to Eq. (1), which insures that *P*_*EXCHANGE *_>*P*_*RETAIN *_if the order of gradients is wrong, i.e. if the gradients of receptor and ligand are antiparallel. By choosing this sign we therefore ensure attraction between axons and ligands.

**Figure 7:**
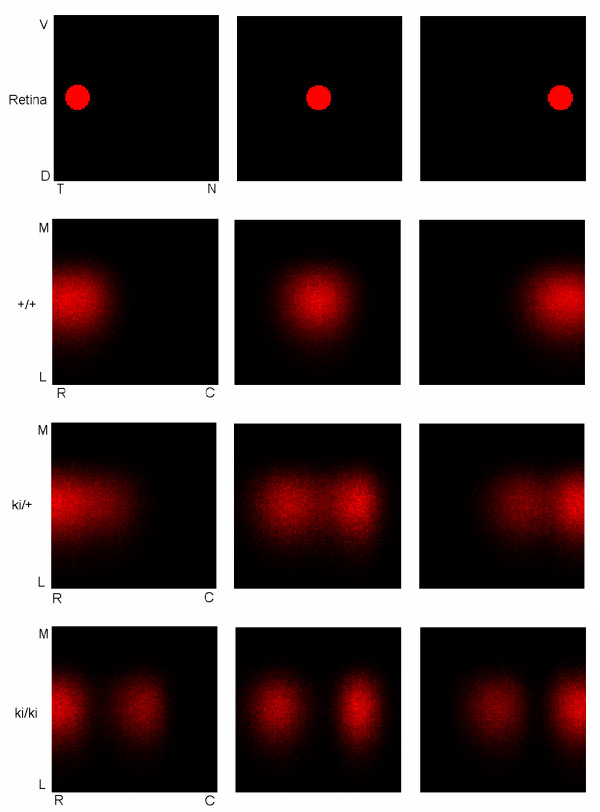
**Numerical simulation of labelling in the 2D model. **The top row shows anterograde "labeling spots" in retina. The following three rows display corresponding distribution of label in SC. The size of both retinal and collicular arrays is 100 by 100 cells. The three rows show results for wild-type, heterozygous knock-ins, and homozygous knock-ins, as marked on the left. Notice the doubling transition, when going from temporal to nasal injection in heterozygotes. This Figure is to be compared to Figure 4 from Ref. [20]. *α *= *β *= 30. The color map is shifted in each image for visibility. Abbreviations: D, dorsal; V, ventral; N, nasal; T, temporal; C, caudal; R, rostral; L, lateral; M, medial.

**Figure 8:**
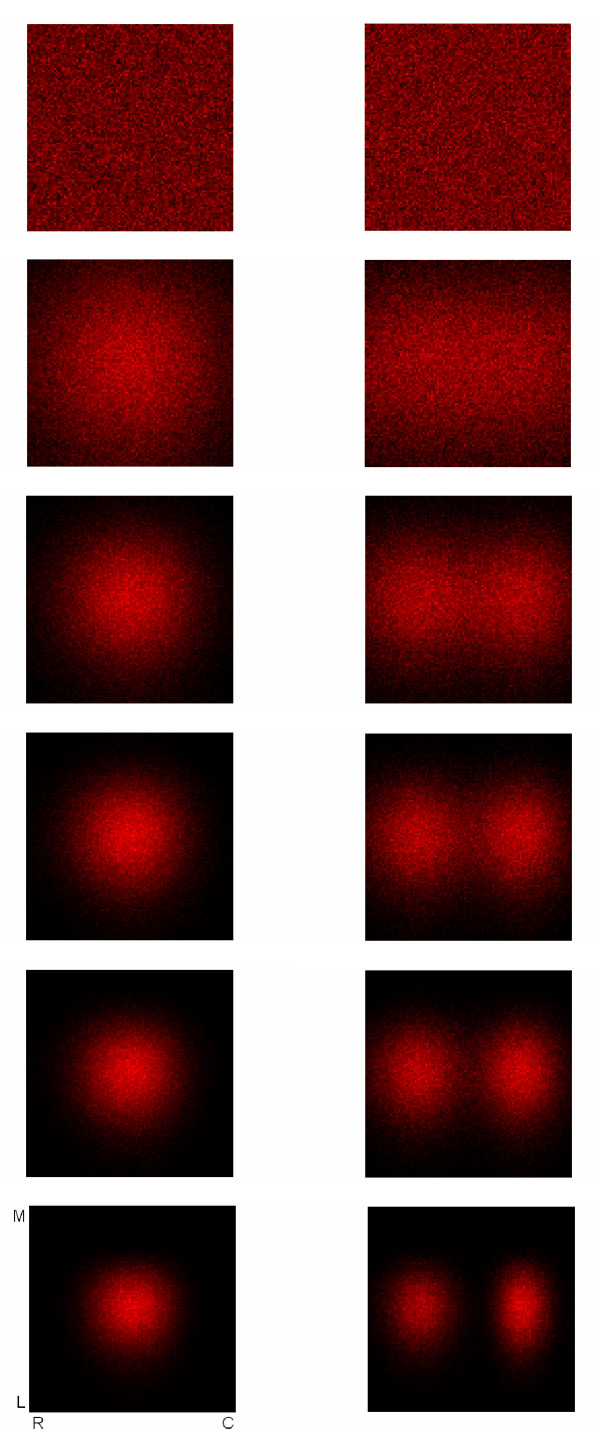
**Map refinement. **Wild-type (left) and ki/ki (right) map development for axons in the central retina. The retinal injection site is the same as in Figure 7, top row and central column. The temporal evolution of the map is shown for t = (0, 8, 16, 24, 32, and 100) × 10000 iterations. The orientation of images is the same as in Figure 7. The color map is rescaled in each image for visibility.

The details of our simulations are described in Methods. Our model allows not only exploration of two-dimensional maps (Figure 7) but also observing and modeling temporal development (Figure 8). Videos with detailed evolution of the map are available in [25].

## Discussion

### Why does the map in heterozygotes bifurcate?

In our model the map is formed through interaction of three factors: Eph/ephrin-based chemorepulsion/attraction, competition between axons for space, and noise. It is the latter that fuses two maps together in rostral SC (Figures 5B) in this model. Therefore, to understand position of the bifurcation point one has to consider the interplay between signal and noise at different positions in the map.

**Figure 9:**
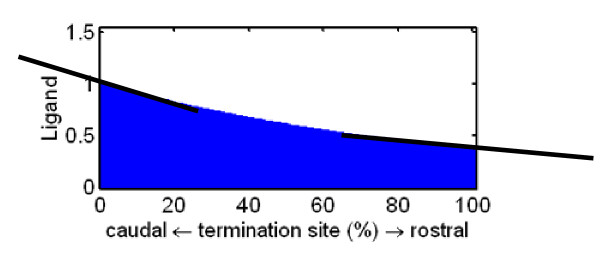
Reduced gradient of ligand in rostral SC may explain single-valued map there.

As we have shown above both ligand and receptor distributions influence the range of single-valued portion of the map independently (Figure 6, columns A and B). Let us first address the impact of ligand distribution. Figures 9 and 12A show that the gradient of ligand is the smallest in rostral SC. This leads to a larger impact of noise there. Since noise drives blending of two branches of the map, this blending first occurs in rostral SC, in agreement with Brown et al. [20]. Interestingly, Brown et al. also shows larger diameters of axonal TZ in the single-valued part of the map, which is consistent with the larger impact of noise there.

**Figure 10:**
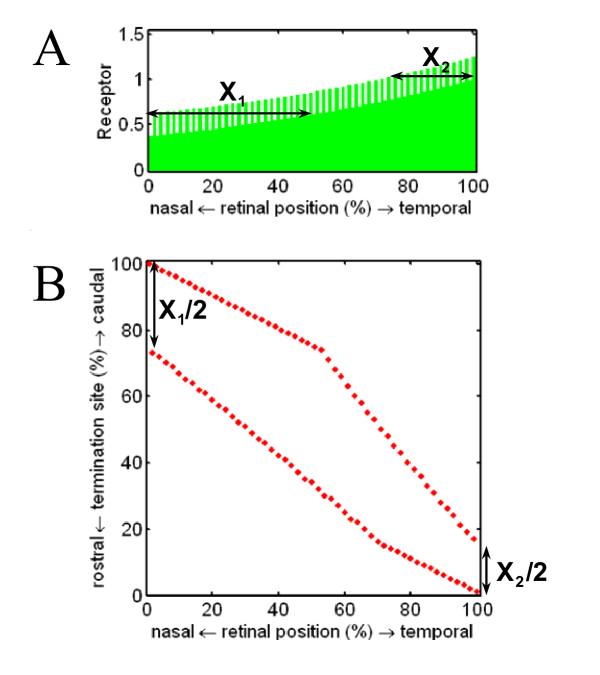
**Mapping for heterozygotes in case of no noise. **The separation between wild-type and EphA3 knock-in axonal terminals is larger in caudal than in rostral SC, due to inhomogeneity in EphA profile.

The second factor contributing to bifurcation in heterozygotes is inhomogeneity of EphA gradient in retina. Consider the case of no noise. Mapping in this case is obtained by sorting axonal terminals in the order of increasing density of EphA (Figures 10, 5, 6). Separation between two maps is the smallest in rostral part (Figure 10B). This is because of inhomogeneous gradient of receptor in 'retinal' cells (Figure 10A). Therefore, even if noise were the same in all parts of the map, rostral part has the smallest signal in terms of separation between two maps, and the largest potential to be blended by noise.

We conclude that two factors, increased noise and reduced signal, cooperate in rostral SC in fusing the wild-type and knock-in maps. This leads to the formation of single-valued map there. In caudal part both noise is reduced and distance between maps is larger. Hence, the map is double-valued in caudal SC.

### Mapping in Isl2/EphB knock-ins has two bifurcations

**Figure 11:**
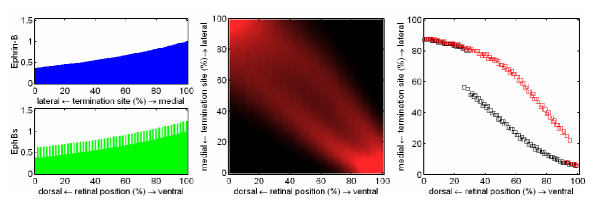
**Mapping in heterozygous Isl2/EphB knock-ins. **Two bifurcations are observed, one in medial, another in lateral SC.

It is possible to spatially separate these two blending factors, increased noise and decreased signal, if one applies the same logic to the DV axis of the map. In our model this mapping is implemented by attractive interactions between EphB+ axons and ephrin-B rich environment. Hence, DV mapping is "flipped" with respect to the TN one in the sense that high EphB gradient region of retina maps to a high ephrin-B gradient region in SC. Two blending effects described above (reduced signal and increased noise) are therefore spatially separated for the DV axis. To observe mapping in these conditions we performed a numerical "experiment" on the Isl2/EphB knock-in conditions. This may have relevance to mapping in DV direction. The results are shown in Figure 11.

Two bifurcations observed in Figure 11 confirm the hypothesis about two factors operating in the numerical model. The ventral bifurcation is associated with receptor, since separation between two maps in perfectly ordered conditions is the smallest in medial SC. The second bifurcation, dorsal, occurs due to noise, since noise is maximal where the gradient of ligand is the smallest, i.e. in lateral SC. Thus, we suggest that experiments on Isl2/EphB knock-ins should make clear if inhomogeneity in receptor density or noise is more important.

It is also possible that activity-dependent mechanisms drive blending of two maps. Activity leads to focusing of projections whereby axons with close locations in retina are effectively attracted to each other in SC. Activity-dependent attraction will blend axons positioned proximally in SC, therefore ventral bifurcation, described above, may be robust with respect to these factors. The dorsal bifurcation, on the other hand, may or may not be observable if activity-dependent focusing of projections takes place. These questions will be addressed in future studies.

### Absolute versus relative

Brown et al. [20] demonstrates that retinocollicular mapping is based on relative levels of EphA/ephrin-A expression in the broad meaning of this term. Indeed, the absolute value of EphA density does not determine where an axon terminates in colliculus. This is because axonal TZ may shift in the presence of axons with altered expression of chemical tags. For example, wild-type axons terminate more caudally in the presence of Isl2+/EphA3+ axons. Hence, an important factor is the presence of other axons, relative to which given axon establishes its termination point. This idea is also evident from retinal and collicular/tectal ablation experiments in rodents [33,34] and other species [2].

Can we take this idea to the next level and hypothesize that relative differences between neighboring retinal cells represent the chemical signal? This suggestion was used [20] to explain blending of the two maps in heterozygous rostral SC, since relative differences in receptor levels are the smallest in the corresponding part of retina (temporal). In this study we present a model, which uses differences in absolute values of chemical label, as seen from Eq. (1). Indeed, in our model adding a constant value to all densities does not change the resulting mapping, since (1) depends only on differences in expression levels. But this manipulation decreases the relative differences in the expression of EphAs between neighboring knock-in and wild-type cells. Hence, our model is not based on relative differences between receptor densities. Yet, we demonstrate that it can account for experimental results in detail. Thus, we suggest that existing experimental evidence is not sufficient to distinguish relative and absolute labeling in the narrower sense.

Of course, our model also accounts for the caudal displacement of wild-type TZs, thus resulting in a relative labeling system in the broad sense. In the first approximation, this model performs a sorting procedure, understood mathematically, of the fibers based on the expression levels of EphA. Our procedure uses differences in absolute values of EphA densities rather than relative differences. We suggest that more quantitative evidence is needed to distinguish these two "relativity principles".

Relative labeling in the narrow sense can be incorporated in our model too, if coefficient *α *is a function of label densities. Thus, the condition *α *∝ 1/(*RA*·*LA*) ensures the Weber's law for axonal "perceptual thresholds", since chemical signal is proportional to the relative differences.

### Comparison to other theoretical models

Theories based on chemoaffinity principle are reviewed in [4]. Some features of our approach are similar to the arrow models described in Ref. [24,27], which employs exchanges between neighbouring axons to establish ordered retinotectal/collicular maps. At the same time some features of our model are different from the arrow model. First, we employ information about chemical labels, Ephs and ephrins. At the heart of our model are equations (1–3), which rely on the known distributions of chemical labels. These equations are unique to our approach. As it was noted above, in the absence of stochasticity (*α *→ ∞), when perfectly ordered map is formed, our 1D model with nearest neighbor exchanges is equivalent to the 1D version of the arrow model. However, in the stochastic regime, description of developmental noise is different here. In particular, we relate features of the map, to the distribution of chemical labels. We argue that this feature is important in understanding experiments [20], since distribution of labels determines where TZs fuse to form bifurcations. Second, we consider both nearest neighbour and distant neighbour exchanges (see Methods for more detail). Indeed, Eq. (1–3) can be applied to determine exchange probability for a pair of distant axons too. This feature may be crucial, since development of map in the RC direction is determined by original primary axonal overshoot with subsequent retraction of inappropriate projections [3,9]. In this process the interstitial branches of the same primary axon are eliminated and subsequently added non-locally. We show below in Method section that in many cases local and global exchanges produce the same results in terms of final distribution of projections. But, the process of development is different in the local and global exchange cases.

Prestige and Willshaw [35] suggested to divide developmental mechanisms into two groups. In group I mechanisms each RGC axon has maximum affinity to certain unique point in the target, even without other axons. In group II mechanisms, the position of TZ results from competitive interactions with other axons. Our model definitely belongs to the second group, since we assume that all axons experience maximum affinity to rostral medial SC and are spread over entire SC by competition. Our approach is similar to described in Prestige and Willshaw in the way how graded distributions of molecular tags are represented. The details of map modifications are somewhat different in this study and are precisely defined by Eqs. (1–3).

In a recent study Honda [36] considered results of experiments [20]. He used servomechanism model to explain the overall structure of the maps in mutants. Servomechanism model is a hybrid between group I and II models in terminology of Prestige and Willshaw, since it assumes that axons have equilibrium points in SC and they are subject to competition with each other. Although Ref. [36] reproduces doubling of the map in homozygotes it does not succeed in obtaining the bifurcation observed in heterozygotes, which is one of the purposes of the present study.

### On the biological realism

When dealing with numerical simulations one always faces the question of the degree of realism with which to model the data. Does one have to model behaviors of individual atoms, or description on the level of axons is sufficient? In this work we choose the level of description on the basis of what is known about this system. We realize that our model does not capture many behaviors, but we argue that the mechanisms involved are unclear at the moment to be incorporated into a more detailed model. Our approach also fulfils its original goal, which is to reproduce the results of experiments [20] and to generate experimentally testable predictions, thus satisfying the requirement of parsimony.

Model presented here does not describe the difference between development along TN and DV axes. The former mapping is controlled by original axonal overshoot along the RC direction in SC, with subsequent elimination of topographically inappropriate projections [3,9]. In contrast, primary axons from the same DV retinal position enter SC in a broad distribution along ML axis. Topographically precise termination is provided by producing additional interstitial branches in the ML direction [31,32,37,38]. These findings cannot be reproduced by our model, since no distinction is made between the primary RGC axon and its branches. Instead, our model deals with terminal points of interstitial branches produced by RGC axons.

## Conclusions

We present a model for retinocollicular map development, which can account intriguing behaviors observed in gain-of-function experiments by Brown et al. [20], including bifurcation in heterozygous Isl2/EphA3 knock-ins. The model is based on chemoaffinity, axonal repulsion/competition, and stochasticity. We discuss possible mappings in ephrinA-/Isl2+/EphA3+ knock-out/ins and Isl2/EphB knock-ins.

## Methods

### 1D model

To find a stationary distribution of the RGC's axons in the SC, we use the following computational procedure. We consider a linear chain of 100 RCG that are connected to one and only terminal cell in SC each. The receptor and ligand expression level profiles used in the computations for wild-type, heterozygote and homozygote are shown at Figure 5A,5B,5C. We start with the random map where the position of every axon in SC does not depend on the level of its receptor expression. Then we perform stochastic reconstructions through an exchange of the positions of the neighboring axons in SC. Namely, at each step we randomly choose one pair of axons out of 99 neighboring pairs and switch their positions with the probability given by Eq. (1). In both cases, whether the positions of the axons are exchanged or they retain at their old locations we proceed to the next step when we choose a new pair of neighboring axons. We repeat the process until a stationary distribution of the probabilities for the positions of the RGC's axons in SC is reached.

The typical stationary solution for one realization is shown at Figure 4. Here the number of iterations is 10^6 ^(nearest neighbor exchanges). The main parameter of our model is taken to be *α *= 30 throughout the paper. It is chosen to fit the experimental data from Brown et al. [20]. We have observed that the value of *α *is roughly equal to inverse of the relative diameter of the TZ squared. Thus, in our results, TZ occupies, roughly, 20% of the entire SC, which corresponds to the value of *α *given above. The probability distribution and the position of the maximums shown at Figure 5 and 6 are obtained by temporal averaging over 5 × 10^4 ^realizations of stationary solution separated in time by 10^3 ^iterations (nearest neighbor exchanges).

### The choice of receptor/ligand expression profiles

**Figure 12:**
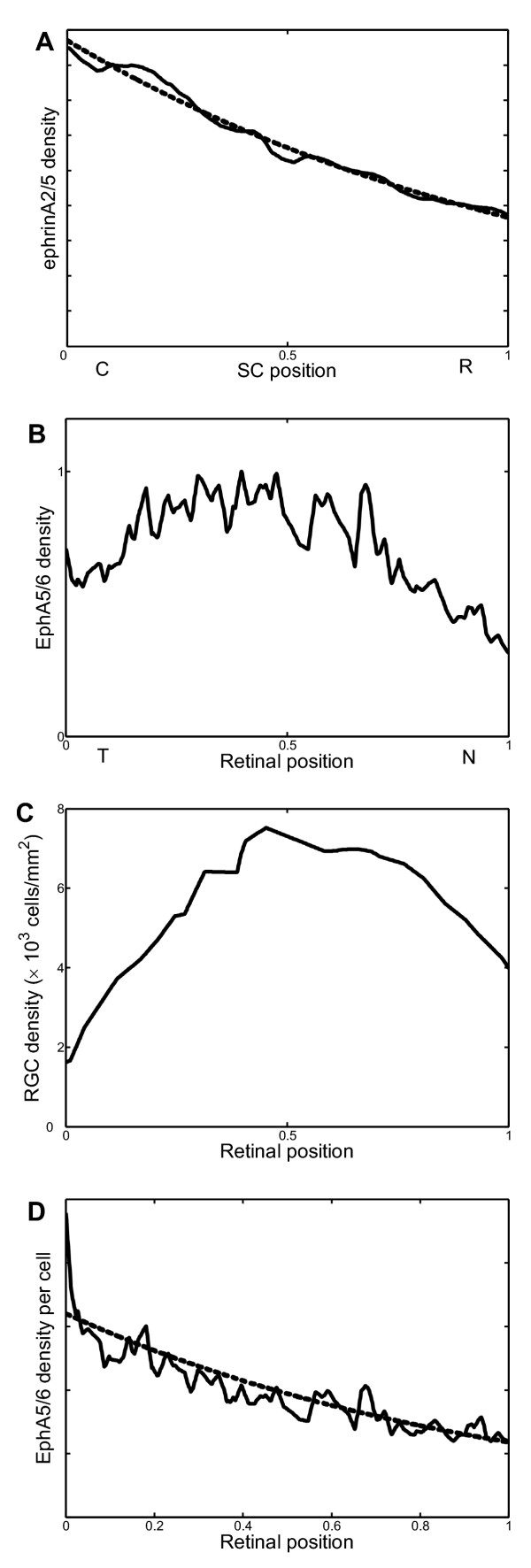
**The choice of receptor/ligand expression profiles. A**, Density of ephrinA ligands in SC obtained from RNA hybridization [10]. B, Density of EphB receptors in retina from RNA hybridization [20]. C, density of RGC in retina [39]. D, Density of EphA receptor per cell, obtained by dividing B by C. Vertical axes in A, B, and D are in arbitrary units. Dashed lines show the exponential approximations used in this study.

We base our choice of parameters for the distribution of molecular markers on experimental observations in mouse retina and SC. Thus, the distribution of ephrinA2 and A5 is obtained in [10]. The total distribution of ligand in SC is shown in Figure 12A. It resembles closely the distribution used in this study *LA*(*x*) = exp(-*x*) (Figure 4A, etc). Note that the constant factor in front of the exponential is taken to be 1 in our model, since any non-unit factor is absorbed into parameter *α *[cf. Eq. (1)].

The distribution of receptors in retina requires more thorough consideration. The distribution of strength of EphA S-RNA hybridization signals is measured in [20] and is shown in Figure 12B. From this distribution one has to obtain the density of receptor expression in single axon, emanating from given point in retina. To this end the overall strength of the hybridization signal is divided by the RGC density in cells/mm^2^, obtained in [39] (Figure 12C). The resulting distribution of EphA receptors per cell is shown in Figure 12D. It is matched closely by the function used in this study *RA*(*x*) = exp(-*x*) (see Figure 4A, etc) in > 95% of retina. Additional distortions introduced by non-uniform linear magnification factor are estimated by us to be small (< 10%), based on data from [39,40]. Such distortions cannot be calculated directly, since a complete topographic map from retina to SC is not available. The errors introduced by non-uniform map do not exceed the precision with which the density of receptors is originally measured, estimated from the noise in [20].

In the EphA3+ retina the density of receptor is increased in every second cell by 50% and 25% of the maximum value in homo and heterozygotes respectively. These parameters are chosen to match the overall map structure (Figure 5) to that observed experimentally in [20] (Figure 2). The particular parameter which was chosen for such comparison was the overall distance between the wild-type and knock-in cells, equal approximately to 40 and 20 percent in homo and heterozygotes. Since such distance is approximately constant in the homozygotes, the effects of receptor dimerization, discussed in [7], are assumed to be negligible. This may occur due to saturated conditions (almost all receptors are in the dimerized state). The effects of ligand dimerization are impossible to estimate at the moment. To assess this effect in our model we verify that our results are not changes significantly if ligand density is below dissociation density for dimerization, i.e. the effective ligand density interacting with the receptor is equal to the square of actual density, *LA*(*x*) = exp(-2*x*) (Figure 6C).

The profiles of expression of EphB/ephrinB pair are measured in [31] similar to EphA/ephrinA. They are taken to be *LB*(*y*) = exp(-*y*) and *RB*(*y*) = exp(-*y*). As with the EphA/ephrinA the non-unit overall factors in these distributions are absorbed in parameter *β *(see below).

### 2D model

Here we describe our 2D model in more detail. We consider an array of 100 by 100 RGC, which are connected to 100 by 100 different points in colliculus. Each RGC is characterized by two levels of expression for two receptors, EphAs and EphBs, described in the text. The concentration profiles are taken to be the same for EphA and EphB receptors in the wild-type species. In the homozygote and heterozygote cases the concentration of EphA is taken as shown at Figure 5, while the concentration of EphB is unchanged. RGCs do not express ligand in our model. The collicular receptacles are described by two ligand concentrations with the same profiles as shown at Figure 5 but with different gradient directions discussed in the text.

The process of development is modeled as follows. We randomly choose a pair of axons in SC separated either in RC or in ML direction. We exchange their positions with the probability given by Eq. (1) or Eq. (3) respectively. We then repeat the process until a stationary distribution of probabilities is reached in the same manner as for 1D case. Note, that this time a chosen pair of axons, say in RC direction, may not be a neighboring pair, but consist of two axons separated by any distance in SC. This procedure dramatically decreases the convergence time to the stationary distribution, which is the same as in the case when we choose the neighboring axons only. The noise level is taken to be the same for both RC and ML directions, that is *α *= *β *= 30.

The spatial 2D distribution of the axons corresponding to labeled RGCs is shown at Figure 7. The "labeling spot" in retina is a circle with radius R = 7.3, the coordinates of the center are (15,50), (50,50) and (85,50) on the 100 × 100 grid. The distribution is obtained by averaging the positions of the labeled axons in SC over 1000 realizations after it reached the stationary solution at 1 × 10^6 ^iterations. The temporal evolution of the map for the label in the central retina is shown at the Figure 8. It corresponds to averaging over 1000 different realizations at each time interval.

In both 1D and 2D cases the calculations were performed on Dell PowerEdge 1600SC server. The programs, written on Matlab (MathWorks, Inc.), are available for download in [25].

### Limiting probabilities between 0 and 1

Equations for the probability of switching of two axons (1) and (2) can yield probabilities, which are below 0 or larger than 1. Thus, in the numerical implementation of our model instead of (1) and (2) we use expressions with soft cutoff at 0 and 1, i.e.


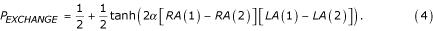



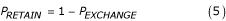


These probabilities are restricted to be between 0 and 1. In addition, when differences in ligand and receptor densities between neighboring points are not large, (4) and (5) are equivalent to (1) and (2).

### Local versus global transitions

One could use exchanges between nearest neighbors to implement map development, as described in text above and in [24]. Alternatively, one could consider swaps between two distant axons chosen randomly. An exact statement, a proof of which we provide here, is that the final probability distribution for connectivities does not depend on whether the swaps are local or global. This statement is true, for any distribution of chemical labels, in 1 or 2D. It is true however if Eq. (4) is used to calculate probabilities of transitions. In particular, we show that Eq. (4) leads to a Boltzmann distribution of the probabilities of connections, which does not depend on the locality/globality of transitions. Thus, we can present final maps for both local and global transitions interchangingly, since results pertaining to the final state of the map, such as in Figures 4,5,6,7 and 10,11, do not depend on the choice of transitions. However, the temporal dynamics of map evolution does depend on this choice. Normally, the convergence of the map to the final distribution is faster with global transitions. Thus, Figure 8 shows evolution of the map for the case of global swaps. The sequence in Figure 8 would be different, if the swaps between nearest neighbors were used.

Let us now derive the probability distribution of projections in the final map. We perform our derivation for the 1D case; in 2D it is similar. We proceed using the detailed equilibrium principle, frequently employed in statistical mechanics [41]. Consider two states of the map, symbolically denoted by A and B. These states are described by corresponding probabilities *P*_*A *_and *P*_*B*_. These probabilities satisfy the detailed equilibrium condition [41]


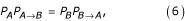


where the transition probabilities are given by equation (4). After some algebra, it is possible to show that the transition probabilities are given by a simpler than (4) form


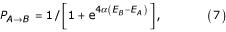


where *E*_*A *_and *E*_*B *_are 'state' variables, depending on the current arrangement of axons in the target


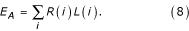


Here summation is assumed over all termination sites in SC, denoted by index *i*, with *L*(*i*) being the ligand concentration and *R*(*i*) the receptor concentration. The latter, of course, depends on the arrangement of axons, corresponding to the state A. The transition probability *P*_*B *→ *A *_is given by the same expression, with exchanged indexes A and B. The detailed equilibrium condition (6) leads then to Botzmann probability distribution of the states of the map


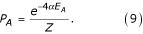


Eq. (9) is instrumental in showing that the final distribution of projections in our approach does not depend on the methods of reconstruction. Thus, both global and local transitions will lead to identical final arrangement of the map. This property is well-known in considering Metropolis Monte-Carlo procedures. What does depend on the methods of reconstruction is the time, which it takes to reach the final configuration. Thus, as it was mentioned above, global transitions lead to the final state much faster. With local transitions, on the other hand the map can freeze in the original state, and it may take an exponential time to reach the final configuration.

We thus conclude that our results presented in this study are universal in that they do not depend on the exact developmental mechanism, but only on the distribution of 'chemical' tags.
